# Errors or Adaptations? A Critical Review of Predictive Processing in Psychiatry

**DOI:** 10.3390/bs16071116

**Published:** 2026-07-03

**Authors:** Matthew Crippen

**Affiliations:** Department of Global Studies, College of Economics and International Trade, Pusan National University, Busandaehag-ro 63beon-gil 2, Busan 43241, Republic of Korea; matthewjcrippen@gmail.com

**Keywords:** ADHD, autism, computational psychiatry and predictive processing, cultural variation, depression, epistemic normativity, PDST, prediction error, schizophrenia, self and agency

## Abstract

Predictive processing (PP) accounts often characterize mental illness as maladaptive and epistemically distorting due to mismatches between brain-generated top-down models and bottom-up sensory inputs, with this review identifying exceptions. First, hypervigilance in trauma survivors with PTSD or depression may sustain desirable gaps between anticipated problems and actual harms that would otherwise occur. Second, PP defenders have argued that depressive slowdowns follow from maladaptive brain-based regulatory models, yet physiological problems may make activity strenuous—in which case slowing down is adaptive. Third, PP researchers introduce tacit normative assumptions. For example, in autism and ADHD, they stipulate thresholds for how specific (hence error-prone) predictive models should be, and PP interpretations of schizophrenia sometimes presuppose Western concepts of self as normative neurocognitive ideals. Fourth, PP accounts of prediction error can tacitly invoke veridical representation, even though advocates regularly claim that cognition evolved primarily for action, not truth-seeking. While criticizing PP for its overreaches, this review also explores how greater attention to these exceptions and factors such as cultural variability may strengthen the framework’s capacity to understand and contribute to the treatment of a range of psychiatric conditions.

## 1. Introduction

Predictive processing (PP) holds that the brain operates through hierarchical inference (A. Clark, 2016; Friston, 2010; Parr & Friston, 2019; Hohwy, 2013). The core hypothesis is that the nervous system builds predictive models from statistical regularities in prior experience (Aitchison & Lengyel, 2017; de Lange et al., 2018); and that organisms perceive, cognize or act according to these priors rather than directly from sensory input, unless incoming signals significantly conflict with and thereby update expectations (A. Clark, 2013, 2016; Friston, 2010; Gordon et al., 2017; Hohwy, 2013; Nemati et al., 2025; Rao & Ballard, 1999; Shao & Beck, 2024).

Regarding mental disorders, PP theorists have proposed mechanisms such as unrealistically negative or positive priors, both increasing prediction error rates (Pellicano & Burr, 2012; Kirihara et al., 2020; Shaw et al., 2020; Sterzer et al., 2018; Van de Cruys et al., 2014). This is along with aberrant precision weightings—“precision” defined as the relative trust placed in sensory input versus prior expectations (R. Smith et al., 2020; Y. Wang et al., 2025). For example, when fog degrades incoming signals, the brain may rely heavily on a preexisting model that makes an obscure figure look threatening or makes intermittently visible lines appear as a road edge (A. Clark, 2013; Hohwy, 2013; de Lange et al., 2018; Rao & Ballard, 1999; Sterzer et al., 2018). In PP nomenclature, this top-down modeling exerts higher- or lower-level effects on conceptual and sensory processing. Whether this is healthy, according to certain PP frameworks (e.g., Kaye & Krystal, 2020; Sterzer et al., 2018), depends on the predictive reliability of the specific prior—that is, its plausibility and helpfulness, otherwise termed epistemic warrant and adaptiveness.

Hence, PP-based psychiatry often regards large, persistent prediction errors as pathological (Adams et al., 2013a, 2013b; Barrett et al., 2016; Fabry, 2020; Kube et al., 2020a, 2020b; Linson & Friston, 2019; Pellicano & Burr, 2012; Putica & Agathos, 2024; Shaw et al., 2020; Sterzer et al., 2018; Van de Cruys et al., 2014; Wilkinson et al., 2017). PP tends also to assume statistically typical predictive profiles, interpreting deviations as neurocomputational failures of inference (Adams et al., 2013a, 2013b; Aloi et al., 2024; Pellicano & Burr, 2012; Pugliese et al., 2022, 2024; Shaw et al., 2020; R. Smith et al., 2020; Sterzer et al., 2018; Van de Cruys et al., 2014). An example is a patient with ADHD who has a narrow concept of a good workspace that even quiet sounds violate.

These PP framings, however, pose several conundrums. A few of these concerns are longstanding in psychiatry, but PP might be expected to do better given the relative newness of the perspective.

First, the notion of statistically typical predictive profiles gets undercut by individual, cultural, historical, material and other differences. Eisenberg et al. (2008), for instance, observe that Ariaal nomads in Kenya displaying ADHD-associated traits fare better on outcomes that matter to them, like securing food. Does this mean that a statistically optimal profile becomes pathological when a nomad relocates to an industrialized environment, only for that individual to be “cured” upon returning home (Crippen, 2025d)?

Second, hardship and danger may not be anomalies but enduring features of environments, as in contexts of chronic trauma or social adversity that contribute to depression or PTSD. If so, expectations of harm may motivate actions that avert it, sustaining desirable prediction errors as gaps between anticipatory models and sensory outcomes.

Third, preventative success may be greater when threats are overanticipated, as sometimes observed in PTSD or depression, partly due to neuromodulatory systems associated with vigilance and threat sensitivity (Abler et al., 2007; Bukhbinder & Schulz, 2016; Hendler & Admon, 2016; MacLeod et al., 1997). Here, adversities may fail to materialize precisely because they are strongly anticipated and actively avoided, though this may also increase false alarms.

Now, is a prior necessarily maladaptive and epistemically unattuned if it sustains prediction errors because it prevents anticipated problems by promoting avoidance strategies? If not, then “hypervigilance” or “paranoia” may sometimes unfairly label what is instead reasonable caution. This may remain true even for predictive models that trade higher false alarm rates for better detection of otherwise missed cues.

Occasionally, there may be no trade-off at all, for what PP regards as low-margin-of-error models—which definitionally implies error-proneness—may sometimes straightforwardly reflect heightened acuity or attunement. This will be discussed in relation to PTSD and depression, albeit without denying that the “pathology” label or interventions are ever warranted. PP can also normatively load schizophrenia by treating deviations from Western experiences of self and agency as symptoms. Yet schizophrenia often appears less severe in parts of the Global South (Luhrmann et al., 2015, 2024; Padma, 2014), where understanding of selfhood and agency typically differ, which may contribute to better prognosis (Crippen, 2025d).

A key concept in PP is active inference (Friston et al., 2017; Parr & Friston, 2019), which reduces prediction errors via behaviors or processes that change the world (or sometimes interoceptive conditions) so that sensory inputs conform to prior expectations. However, the defensive scenarios outlined above involve actions that maintain gaps between expected harms and actual outcomes, thereby sustaining desirable prediction errors. Further, there are cases where anticipating failure brings about the predicted outcome (Maier & Seligman, 2016). Although PP theorists might claim that error-prone hyperprecision is part of this process (Barrett et al., 2016; A. Clark, 2016; Friston et al., 2017), it could instead reflect pathology arising from alignment between priors and outcomes.

Another core PP precept is that cognition is an action-guiding tool and that the brain evolved to minimize expected surprise, which does not always entail representing affairs accurately (Bruineberg et al., 2018; A. Clark, 2013, 2016; Facchin, 2021; Friston, 2010, 2011; Kiverstein et al., 2019; Hohwy, 2020; Parr et al., 2022; Schwartenbeck et al., 2015). Here, mental models resemble subway maps that reduce uncertainty without high-fidelity resemblance (Crippen, 2017). One point to accordingly consider is whether the PP idea of mismatches between models and the world contains a hidden commitment to veridical representation. This may bias PP toward pathologizing persistently large prediction errors, overlooking that they can be adaptive and epistemically attuned.

Notice further, however, that many tools (e.g., shovels) facilitate coping without map-like representation (Crippen, 2017; also see James, 1907). Perspectives more in line with this view include enactive cognitive science (de Haan, 2020) and ecological psychology (E. J. Gibson & Pick, 2000), plus their historic antecedents in American functionalism or pragmatism (Dewey, 1896) and phenomenology (Merleau-Ponty, 1945/1962). As with PP, these approaches are action-based but suggest perception need not involve internal representation: water appears walkable or swimmable less from internal modeling and more from strict limits on what a creature can do (E. J. Gibson & Pick, 2000; also see Dewey, 1896; Merleau-Ponty, 1945/1962; Varela et al., 1991).

Extending this logic, moods and emotions affect capacities. As such, objects typically appear farther away to sad individuals (Riener et al., 2011), but this need not reflect distorted modeling or prediction errors. After all, depression involves alterations in interoception, autonomic activity, fronto-striatal coordination and sub-cortical signaling, alongside neuroimmune adjustments such as inflammation—changes that are often fatiguing (Gkintoni et al., 2025; Guan et al., 2024; C.-H. Lee & Giuliani, 2019; Schumann et al., 2025; Tian et al., 2025; Targum & Fava, 2011). Depression accordingly coincides with physiological burden (Eggart et al., 2023; Ghanean et al., 2018; Pastuszak et al., 2025; Schumann et al., 2025; Shinba et al., 2023; Targum & Fava, 2011; Tian et al., 2025). Ongoing physiological strain thus creates conditions where targets are genuinely harder to reach, so that greater perceived distances may index this toll (Crippen, 2025d). Hence, the predictive model—assuming it is at play—may follow from somatic changes rather than causing them, and it need not be inaccurate.

To recapitulate, predictive models may sometimes be consequences rather than causes of somatic burden while remaining predictively accurate. Other times, alleged overanticipation of negative possibilities can be helpful. In wartime, restlessness and rigid interpretation—such as not conjecturing that loud noises may be fireworks—can be life-preserving, even if attacks are statistically infrequent (Crippen, 2025a). Following sexual abuse, anticipatory monitoring may rationally persist given the high risk of re-victimization (see Barnes et al., 2009; Sorenson et al., 1991; H. E. Walker et al., 2019; World Health Organization, 2024). Trauma-induced depression may likewise promote analytic processing and problem anticipation, helping prevent recurrence (Andrews & Thomson, 2009; Andrews et al., 2020). Accordingly, it is worth distinguishing harmful circumstances from adaptive responses and their injurious downstream effects. In addition to subjective suffering, sustained vigilance associates with neurobiological changes such as dopaminergic irregularities and brain atrophy in certain regions (L. L. Chao, 2016; McEwen, 2005).

In many cases, therefore, PP advocates—and clinicians more generally—appropriately advise treatment. Still, the overall account puts pressure on PP. First, the physiological insults arising, for example, from worry that generates prediction errors by helping prevent adverse outcomes may compare to injuries sustained through adaptive behavior, such as tearing a muscle while escaping a rabid dog. Second, some PP defenders advance broad unifying claims (A. Clark, 2013, 2016; Friston, 2010; Hohwy, 2013). This makes even a small number of counterexamples problematic. Third, PP already faces difficulties securing strong empirical confirmation, especially neurobiologically (Bowers & Davis, 2012; Hodson, 2024; Miłkowski & Litwin, 2022; Qela et al., 2025). If psychiatric conditions can arise from both predictively accurate and inaccurate models, and if these models can function as either causes or consequences of disorder, then the falsifiability of PP is weakened.

Simultaneously, this review highlights compelling sides of PP. While predictive models are not directly detectable through neuroimaging, existing brain science fits many PP claims about mental illness. PP framings can also align with the phenomenology of mental illness. Further, PP may gain explanatory power by attending more to certain individual differences. For example, lower agency appears more associated with schizophrenia in some regions than others. A PP framework that accounts for variation in culturally conditioned priors may improve understanding and diagnosis of schizophrenia.

It is a truism that mind and brain are complex, arguably more so than what is observed in physics, which remains ununified. For understanding and treating mental illness, pluralism may be fruitful. This can mean questioning the grand unifying overreaches of PP and the anti-representationalism of certain ecological and enactive strains. Given the health stakes, it may be wise to put ideological commitments aside, even drawing on conflicting frameworks.

## 2. Anticipatory Processes in Action-Oriented Cognition

Several themes recur throughout this article concerning the integration of affect, cognition, action and perception, both in ordinary and clinical contexts, and this section summarizes them. The aim is to clarify commitments that inform later criticisms of PP.

Western commentators have historically portrayed emotions as epistemic contaminants, though exceptions exist (for review, see Crippen & Schulkin, 2020; Loewenstein, 1994). Since the 1990s, however, neurobiologists and cognitive scientists such as Schulkin (Parrott & Schulkin, 1993) and Damasio (1994) have emphasized emotional dimensions of rational choice, sustained effort and selective attention, citing functional psychology and pragmatism as inspirations. Gigerenzer (2007) makes similar points. Pessoa’s (2013) integrative framework further dissolves sharp boundaries between cognitive and emotional systems at the neural level.

Together, these strands establish a conceptual and empirical backdrop for evaluating PP psychiatric interpretations since emotional disturbances often characterize mental illness. A more PP-oriented consideration is that affective processes regulate uncertainty-reducing cognitive and perceptual activity, narrowing attention and excluding irrelevant information (Damasio, 1994; Jacobs et al., 2012; Parrott & Schulkin, 1993; Pessoa, 2013).

Neurobiological evidence ties affectivity to cognitive functioning. The amygdalae, for instance, are implicated in selective attention and assigning value to perception and cognition (Pessoa, 2013). Amygdalar circuits couple closely with hippocampal and cortical systems, together supporting memory consolidation, language capacities and concept deployment (Babaev et al., 2018; Citron et al., 2020; Grupe & Nitschke, 2013; Grupe et al., 2013; Jacobs et al., 2012; Šimić et al., 2021; Tyng et al., 2017). Epistemically complex emotions, including shame, envy and embarrassment, likewise recruit distributed cortical and subcortical networks involved in semantic processing and social cognition (Bastin et al., 2016; Binder et al., 2009; Jankowski & Takahashi, 2014).

Another example is the insular cortices, associated with emotions such as empathy, fear and disgust (Cantone et al., 2019; Uddin et al., 2017), which are again epistemically complex because they guide social behavior and threat appraisal. Through connections with the basal ganglia, insula-centred circuits are implicated in habit learning, reward evaluation and action selection (Hikosaka et al., 2014). Cortico–basal ganglia networks also support the temporal and syntactic interpretations of language and music, domains that depend on anticipation and expectation (Grahn & Rowe, 2009; Kotz & Schmidt-Kassow, 2015; Kotz et al., 2009).

Everyday experience further illustrates the entwinement of affect, cognition, perception and action (Crippen, 2022). Objects and situations acquire significance relative to affectively shaped goals; superficially similar linguistic expressions convey different meanings depending on expressive tone; and emotions modulate attention, associative processes and memory (Dewey, 1925; Heidegger, 1927/1962; James, 1884, 1890; Johnson, 2007; Luria, 1968). Affective interests additionally influence concept formation, as illustrated by the differing meanings of oil for a carpenter, a mechanic and a dry cleaner (James, 1879b; also see Barrett et al., 2016; Damasio, 1994; de Sousa, 1987; Parrott & Schulkin, 1993). In line with this, affective neural systems, including amygdalar circuits, are involved in both object recognition and action readiness (Pessoa, 2013).

Affectivity accordingly shapes perception by modulating sensitivity to possibilities for action (Crippen, 2022). Consider a hiker engaged in skilled, purposeful movement across a frozen lake. She might perceive stable and fragile portions as emotionally safe or dangerous, thus as either affording or not affording locomotion (Crippen, 2017). One upshot is that affordances—as conceived in ecological psychology—are inherently prospective, since perceiving what can be done requires sensitivity to outcomes that have not yet occurred.

While PP conflicts with ecological psychology by presupposing internal brain-based models that construct experience of the world, PP obviously shares ecological psychology’s prospective slant. On a PP account, the hiker, if walking on an urban sidewalk, would not register the absence of open utility holes or loose concrete tiles; she would instead rely on predictions of easy passage, unless interrupted by an unexpected hazard, with PP advocates arguing that this enhances efficiency (Aitchison & Lengyel, 2017; A. Clark, 2013, 2016; Friston, 2010; Hohwy, 2013; de Lange et al., 2018; Parr & Friston, 2019; Rao & Ballard, 1999; Shao & Beck, 2024).

Emotions such as worry and hope involve expectations. For PP advocates and non-advocates alike, affectivity is central to anticipation (Bar, 2009; Barrett, 2017a, 2017b; Hoemann et al., 2017; K. M. Lee et al., 2021; Marroquín et al., 2016; Ridderinkhof, 2017; Stefanova et al., 2020; Šimić et al., 2021). Emotional anticipation, moreover, is almost definitionally cognitive insofar as it forecasts what is not present. This dynamic is evident in language. For instance, “but” and “and” are logically identical; yet listeners typically anticipate different emotional closures to the statements “I love you but…” and “I love you and…” (Crippen, 2025d). Similarly, the phrase “in the event” constrains expectations to anticipate continuations like “that” or “of,” yet not “pie” or “to” (Weaver, 1949). This last example comes from a pioneering book in information theory that basically advances the PP contention that predictive models enhance efficiency and reduce uncertainty.

These patterns therefore align with the PP proposition that cognition involves the generation of expectations about future sensory states (den Ouden et al., 2012; Feldman & Friston, 2010; Parr & Friston, 2019). In this framework, affectivity modulates both the content and the relative weighting of the generative models that guide perception and action (Auksztulewicz & Friston, 2016; Barrett et al., 2016; Barrett, 2017b; Demekas et al., 2020; Seth, 2013; Seth & Friston, 2016; R. Smith et al., 2020). Emotions, interests and moods direct attention toward task-relevant information, modelling the world by shaping expectations about action–outcome contingencies while regulating sensitivity to prediction error (Auksztulewicz & Friston, 2016; Demekas et al., 2020; den Ouden et al., 2012; Friston et al., 2017; Parr & Friston, 2019).

Relevant to PP and active inference, dorsal striatal circuits support habit formation, which is modulated by affective and motivational systems like the amygdala bulbs (Giovanniello et al., 2025; Graybiel, 2008; Lingawi & Balleine, 2012; Schwabe & Wolf, 2009, 2013; Wirz et al., 2018; Yin & Knowlton, 2006). Habits improve efficiency by constraining action selection, reducing uncertainty, increasing focus and lessening attentional demands (Dewey, 1922; Graybiel, 2008), thereby modelling perception. PP accordingly characterizes habitual programs as higher-level priors, updated when unanticipated outcomes result in prediction errors (Friston et al., 2017; Parr & Friston, 2019; Proietti et al., 2025; Schwartenbeck et al., 2013; R. Smith et al., 2019c, 2022).

Whether specifically advancing a PP standpoint or not, bodily activity scaffolds psychological processes. Posture influences arousal, cardiovascular activity and neural processing (Muehlhan et al., 2014; Riskind & Gotay, 1982), while widening or squinting eye movements associated with surprised fear and disgust differentially support stimulus localization or discrimination (D. H. Lee et al., 2014). Cultural habits further shape affordance perception, influencing what is perceived as edible, familiar, or aversive within a given environment (J. J. Gibson, 1979).

The discussion of habits bears on PP accounts about the calibration of precisions. As an illustration, consider the rarity of jaywalking in Tokyo, which may stabilize higher-level priors that guide behavior, allowing people to mostly ignore pedestrian signals and automobiles and instead simply follow others, crossing when they do (Crippen, 2025a). Here, behavioral cues carry high precision—that is, the expected probability of prediction error is low. For this reason, certain perceptual inputs (pedestrian signals, cars) remain backgrounded until, say, a non-conforming foreigner violates established conventions. However, if a Tokyoite relocates to Cairo, where crosswalk norms are mostly absent, repeated prediction errors will ordinarily update the prior. Put plainly, the movement of others will cease to be a dependable indicator, with precision weighting accordingly readjusting (Crippen, 2025a).

Now, retaining a Japanese jaywalking prior after permanently relocating to Cairo would be maladaptive. PP theorists interpret mental disorders as structurally similar. Psychopathology is framed as a disturbance in the balance between prior expectations and sensory evidence, particularly through aberrant precision weighting—that is, misestimating the relative reliability of priors and incoming signals (Adams et al., 2013b; Friston, 2010; Hohwy, 2013; Parr & Friston, 2019; Sterzer et al., 2018).

Building on this, the next section discusses a recurrent PP hypothesis: certain psychiatric conditions are marked by excessively precise (narrow) or rigid higher-level priors that fail to update appropriately, thereby sustaining maladaptive expectations over time.

## 3. Precision, Prediction and Psychopathology

This section provides an overview of PP accounts of psychopathology. Subsequent discussions examine some disorders in greater detail and critically evaluate PP framings.

On PP accounts, mental health conditions may arise when higher-level priors become overly rigid or precise, limiting belief updating in response to environmental input (Adams et al., 2013b; Sterzer et al., 2018). Another issue can be overly precise thresholds for sensory prediction errors (Aitchison & Lengyel, 2017; A. Clark, 2016; den Ouden et al., 2012; Feldman & Friston, 2010; Friston, 2010; Hohwy, 2013; Pellicano & Burr, 2012; Van de Cruys et al., 2014). For example, the faint sound of a tennis ball bouncing on a court ten stories below a library room would not violate most expectations of a quiet workspace. However, a student with ADHD or autism may be exacting, thereby registering a gap (error) between the situation and their expectations (Pellicano & Burr, 2012; Van de Cruys et al., 2014).

For PTSD, a PP proposition is that trauma establishes hyperprecise threat-related priors; these dominate perceptual inference, biasing ambiguous cues toward danger, impairing extinction or safety learning (Kube et al., 2020a; Linson & Friston, 2019; Linson et al., 2020; Pierce & Black, 2024; Wilkinson et al., 2017).

Disrupted precision weighting, similarly, is hypothesized to underlie hallucinations and delusions, with psychosis involving higher-order beliefs that resist counterevidence (Adams et al., 2013b; Corlett et al., 2016; Sterzer et al., 2018). To illustrate, PP literature discusses how experimenters led participants to expect the song “White Christmas.” White noise was played instead, with a percentage nonetheless reporting hearing the song (Corlett et al., 2019; Friston, 2010; Linson & Friston, 2019). Another proposed mechanism is over-attunement to minor sensory prediction errors (A. Clark, 2016; den Ouden et al., 2012; Feldman & Friston, 2010; Friston, 2010; Hohwy, 2013; Pellicano & Burr, 2012; Van de Cruys et al., 2014).

A paranoid patient might register minute divergences from behavioral norms as indicating another’s bad intent, even though these deviations pass unnoticed by most. Or minor statistical noise—say, wavering shadows produced by swaying curtains filtering moonlight—might violate an “all-safe” predictive model, leading the individual to infer the presence of an intruder.

Trauma may heighten receptivity to certain sensory inputs, with an interviewee from one study reporting that she can smell cigarettes on those who have brushed their teeth and showered because, during her childhood, the odor signaled an abuser’s proximity (Crippen, 2025d). Under Paulus and Stein’s (2006) interpretation, an initially neutral stimulus—tobacco—becomes conditioned as a threat cue, generating interoceptive signals such as elevated pulse and internal shakiness. This response can persist even after the danger has passed. Paulus and Stein add that in anxiety-prone individuals, the anticipatory signal is excessively precise. That is, the brain emphasizes low-grade differences between expected and actual arousal, such that worry and behavioral avoidance are easily triggered.

PP sometimes characterizes such dynamics in terms of active inference, in which agents act to bring sensory inputs, including interoceptive ones, into alignment with their predictions (Seth, 2013). When struggling with volatile environments, the brain might shift into a “safety first” mode, selecting actions that minimize the likelihood of unpleasant surprises. This is active inference, and it may provide short-term relief while preventing the person from learning that the situation is safe (McGovern et al., 2022).

Relevant PP framings of mental illness are consistent with cognitive penetrability, understood as the idea that higher-level cognitive states—such as beliefs, desires or intentions—can directly shape perceptual experience (Marchi & Newen, 2015). For example, when riding a subway, a person may mistakenly reverse the east–west orientation of the windows, producing the impression of moving in the opposite direction (Crippen, 2025a). Hypothetically, one could have a prior in which east and west are constantly inverted, thereby creating a persistent hallucination of travelling opposite the train’s actual trajectory.

PP models of psychosis extend this logic. On influential accounts, delusions and certain symptoms of schizophrenia arise when aberrant precision weighting disturbs the balance between prior expectations and sensory evidence (Adams et al., 2013b; Corlett et al., 2009; Sterzer et al., 2018). Highly precise priors may resist revision despite prediction errors (Adams et al., 2013b; Fletcher & Frith, 2009; Sterzer et al., 2018). Meanwhile, overweighted sensory errors can drive unstable updating and implausible hypotheses to explain noisy inputs (Corlett et al., 2016; Corlett & Fletcher, 2010; Hauke et al., 2022; Sterzer et al., 2018). Examples of such occurrences have already been given.

PP also proposes overly imprecise priors as contributors to schizophrenia (Ford & Mathalon, 2012; Y. Huang & Rao, 2011; Liddle & Liddle, 2022). Here, the brain may have a weak prior predicting silence in a room devoid of people, televisions or other sources of vocalization, thereby increasing the likelihood that internal thoughts and dialogues are misattributed to an external source. PP theorists also propose that patients could have weak models for differentiating self-produced internal speech, construing it as coming from without (Ford & Mathalon, 2012). Psychotic symptoms are thus framed not as wholly sui generis phenomena but as extreme variants of the same inferential mechanisms that ordinarily support adaptive perception and action (Fletcher & Frith, 2009; Powers et al., 2017; Sterzer et al., 2018).

In depression, negatively biased and overly stable priors about the self or the future may blunt responsiveness to favorable prediction errors, sustaining pessimism (Kube et al., 2020b). Or the disorder can arise when the brain deploys models that poorly govern internal allostatic processes (Barrett et al., 2016). One possible causal contributor is dysregulated release of cortisol and corticotropin-releasing hormone (CRH), which may diminish energy when it is needed or elevate it when it is not (Crippen & Schulkin, 2020; Crippen, 2025a), contributing to cycles of depressive slowing and mania.

This last example provides a convenient way to illustrate both overlap and divergence between PP and other action-based interpretations, particularly those from ecological psychology (e.g., Proffitt, 2006; Witt, 2011). Emotions like sadness can reduce energy and action capacity, making stairs and hills appear farther away or steeper, that is, more effortful to reach (Proffitt et al., 2003; Riener et al., 2011; Schnall et al., 2010; Stefanucci et al., 2008). This seems ecologically attuned. Stimuli (people, in this case) can also appear closer when perceived as threatening or disgusting (Cole et al., 2013). The effect may be tied to regulatory processes (e.g., adrenaline elevation) that increase available energy and may serve an epistemically adaptive function by keeping potential dangers in focus.

On this ecological view—which here closely parallels strands of pragmatism, phenomenology and enactivism—there is little misrepresentation or aberrant modeling in the first place. Diminished vitality does not distort perception so much as disclose a genuinely more demanding world (Crippen, 2025b, 2026a). This is especially so if one considers that units such as meters are a relatively recent historical imposition, whereas the difficulty of reaching a target may be a more fundamental measure of distance, whether for an ancient hunting party or a tired student struggling to get home.

From standard PP perspectives, however, these perceptual changes reflect epistemically disordered predictive modeling. This distorts perception and lowers expectations of success, heightening anticipated energy expenditure and contributing to allostatic dysregulation (Arnaldo et al., 2022; Barrett, 2017a; Barrett et al., 2016; Barrett & Simmons, 2015; J. E. Clark et al., 2018; Davey, 2025; Fabry, 2020; Kube et al., 2020b; Pizzolla, 2026). By contrast, ecological psychology, enactive cognitive science and their antecedents in pragmatist, functionalist and phenomenological traditions stress how reduced energy reshapes an agent’s capacity to cope with environmental demands (see Crippen, 2025d, 2025a, 2025b; Crippen & Schulkin, 2020).

In sum, PP usually prioritizes brain-generated models or representations as the central determinants of experience (Barrett, 2017a; Ongaro & Kaptchuk, 2019; Wilkinson et al., 2017). Pragmatists, phenomenologists, ecological psychologists and enactivists place greater emphasis on body–environment interactions, treating them as constitutive of experience itself (de Haan, 2020; Dewey, 1896, 1920; E. J. Gibson & Pick, 2000; Merleau-Ponty, 1945/1962; Varela et al., 1991; for review see Crippen, 2025b). Commentators from these camps often avoid talking about mental representations or models altogether. To the extent that they invoke them, representations are typically understood as consequences rather than antecedents of experience in the sense just defined. As subsequent sections will discuss, this second theoretical position highlights normative impositions tacitly inserted into PP interpretations of a range of mental illnesses.

## 4. Depression, Prediction Error and Anticipatory Problem-Solving

PP proposes that depression arises from maladaptive predictive models and aberrant precision weighting, resulting in epistemically distorting prediction-error dynamics. The following critically interrogates this neurocomputational account.

One objection revolves around ruminative worry. Associated with depression and sometimes psychosis in PP and non-PP accounts discussed below, prospective brooding may aid problem-solving by helping individuals anticipate and avert future harm (Crippen, 2025a). In such cases, adaptive and epistemic success may depend on consistently sustaining large prediction errors, understood as discrepancies between feared and actual outcomes. A second objection is that depression can emerge under conditions where alleged distortions instead reflect environmentally calibrated and, in some cases, warranted expectations.

The PP position, however, is not without merit and arguably captures important aspects of depression. A central claim is that depression involves maladaptive top-down models that disrupt interoception or bias attention toward adverse and threat-related information through altered priors and precision-weighting. The result is sustained and self-reinforcing unpleasant feelings, heightened stress and pessimistic expectations (Barrett et al., 2016; Barrett & Simmons, 2015; J. E. Clark et al., 2018; den Ouden et al., 2012; Friston et al., 2014a; Kube et al., 2020b; R. Smith et al., 2019a, 2019b, 2021).

Aligning with this view, empirical work indicates that depression associates with anxiety and bleak past- and future-oriented stances (Grupe & Nitschke, 2013; Kellogg et al., 2020; MacLeod et al., 1997; Strunk et al., 2006). Compared to controls, those with depression exhibit heightened amygdalar activation when expecting negative outcomes or pondering sad events (Abler et al., 2007; MacLeod et al., 1997). Also, non-depressed individuals anticipating aversive stimuli show neural activity resembling the baseline profile of depressed patients (Abler et al., 2007). Within PP, excessively precise (high-confidence) negative priors (beliefs or models held beforehand) become resistant to counterevidence; this may stabilize delusional beliefs of guilt, persecution or nihilism, suggesting depressive and psychotic symptoms may lie along a continuum of maladaptive precision-weighting (Corlett et al., 2009; Friston et al., 2014a, 2014b; Sterzer et al., 2018).

As stated, however, exclusively maladaptive framings of depression warrant qualification. Like relieving a dehydration headache with water, aversive moods may sometimes serve regulatory purposes, motivating corrective action (Nesse, 2000, 2019). Similar patterns appear in non-human animals. In one experiment, squid given local anesthetic after tentacle amputation responded as though uninjured, suffering greater predation than their untreated counterparts (Crook et al., 2014). More broadly, injury and sickness behaviors across species involve safety-oriented, energy-conserving responses (Dantzer et al., 2008; Hart, 1988).

A line of evidence for the preceding view comes from rodent models, which induce a depression analogue via gastrointestinal illness or starvation. In the first case, rats show diminished libido and appetite alongside increased sleepiness and psychomotor slowing (Andrews et al., 2020). These patterns exemplify sickness behavior, a reallocation of physiological resources to conserve energy and facilitate immune response (Andrews et al., 2020; Dantzer et al., 2008). Starvation likewise suppresses sexual behavior but increases motor activity and environmental exploration, facilitating food acquisition (Andrews et al., 2020). Human data from prolonged caloric deprivation likewise reveal marked motivational and behavioral reprioritization (Keys et al., 1950). Together, these findings suggest that different forms of physiological strain elicit at least some adaptive responses.

For some time, commentators have asserted that experiences of adversity can intensify deliberative modes of cognition (Andrews & Thomson, 2009; Andrews et al., 2020; Bartoskova et al., 2018; Crippen, 2022; Csikszentmihalyi & Wong, 1991; De Fruyt et al., 2020; Dewey, 1916, 1925; Elmer & Stadtfeld, 2020; Fazio et al., 2024; Forgas, 2013; Heidegger, 1927/1962; James, 1879a; Kellogg et al., 2020; Nesse, 2000; Nietzsche, 1888/1954; Rottenberg, 2014; Wittmann, 2014). This observation both aligns with and challenges PP claims.

On the one hand, intense and persistent rumination associates with depression as well as clinical and especially subclinical psychosis (Fazio et al., 2024). Noting this, PP theorists attribute psychosis to overly rigid predictive models that persist despite disconfirming sensory evidence (Adams et al., 2013a, 2013b; Arnaldo et al., 2022; Berg et al., 2022; Corlett et al., 2016). Beyond psychosis, PP frameworks have also invoked active inference to explain characteristic features of depression, including psychomotor retardation (Arnaldo et al., 2022; Barrett et al., 2016; Davey, 2025; Davey & Badcock, 2025; Kirchner et al., 2024). Here, for example, the brain may assign excessive precision to small downward fluctuations from expected energy levels, causing them to be interpreted as significant exhaustion. If the person then slows down and behaves in a fatigued manner, sensory inputs fall into alignment with the prior (Paulus & Stein, 2006).

On the other hand, evidence links dysphoria to increased systematic processing (Forgas, 2013), which may contribute to healing (Andrews & Thomson, 2009; Bartoskova et al., 2018). Consistent with this, depression coincides with rumination and behaviors sustaining it (Andrews & Thomson, 2009; Bartoskova et al., 2018). This includes social retreat, though dyadic relationships are sometimes maintained despite withdrawal from larger groups (Elmer & Stadtfeld, 2020). The afflicted may abandon previously rewarding activities, such as sex (Andrews & Thomson, 2009; De Fruyt et al., 2020; Elmer & Stadtfeld, 2020), paralleling the earlier described rodent research (Andrews et al., 2020).

Taking stock of what has been said, a depressive slowdown may follow an unfortunate event; this may help a sufferer reevaluate matters to forestall a recurrence. At least occasionally, then, there may be adaptive value in anticipatory caution and motor retardation (Andrews & Thomson, 2009; Azorin et al., 1995; Caligiuri & Ellwanger, 2000; Karpinski et al., 2018). Enhanced analytic performance under low mood may also confer adaptive advantages (Andrews & Thomson, 2009; Karpinski et al., 2018). This interpretation is reinforced by the finding that dreaming and trauma-focused reflection can hasten recovery (Andrews & Thomson, 2009; Pigeon & Carr, 2016; Wittmann, 2014).

Together, these considerations support the qualified claim that ruminative depressive tendencies may, under constrained conditions, function as an epistemic strategy for resolving personal difficulties. Excessive and rigid forms may nonetheless contribute to maladaptive trajectories across affective and psychotic disturbances. As will later be discussed, depressive rumination can cause brain atrophy, likely through prolonged strain on certain neural systems. Often this warrants treatment, but the physiological insult is here comparable to an injury resulting from adaptive behavior, as when tearing a muscle while fleeing a rabid dog (Crippen, 2025d, 2025a).

In fairness, PP authors occasionally get close to defending adaptive accounts of depression. Badcock et al. (2017, p. 188), for example, talk about a “better safe than sorry” strategy aimed at minimizing expected surprise. On this view, the depressed brain adopts predictive models that reduce social uncertainty when environmental cues signal volatility and greater risk of aversive interpersonal outcomes. While alleviating short-term stress, sustained and elevated risk-averse behavior is not always desirable.

Here, an intervention might reengage individuals in ways that increase the discrepancy between entrenched negative self-representations and positive social input—allowing gradual model revision. This suggests that effective treatment may sometimes involve strategically increasing early prediction error, with implications for staged or early-phase intervention in at-risk populations.

Such considerations reinforce a broader point developed throughout this section: prediction errors associated with depression may sometimes serve adaptive or protective functions rather than reflecting straightforward dysfunction.

## 5. Adaptive and Epistemically Calibrated Prediction Errors

Building on what has been said, this section develops a finer-grained account of how PP explains interoception in depression, focusing on Barrett et al.’s (2016) account. It extends the critique that prediction errors may be adaptive and epistemically calibrated while laying groundwork for the later discussion of psychosis.

Barrett et al. (2016) invoke allostasis to situate depression within a PP framework centered on disruptions in interoceptive regulation. Whereas homeostasis refers to the maintenance of relatively fixed physiological set points (e.g., sodium balance), allostasis concerns regulatory processes with variable optimal ranges. In many species, animals undergo allostatic seasonal changes—winter torpor to conserve energy, heavy eating to store calories and brown fat growth for thermogenesis (Crippen, 2025a; P. Lee et al., 2013; van der Lans et al., 2013).

In humans, these adjustments may occur as an evolutionary carryover, once adaptive but less so in settings with warm clothing, building insulation and heating. If the brain overpredicts environmental cold, the perceptual misrepresentation and associated active inferences may resemble a mild hallucination, along lines discussed by PP advocates (Adams et al., 2013b; Corlett et al., 2009, 2016; Corlett & Fletcher, 2010; Fletcher & Frith, 2009; Powers et al., 2017; Sterzer et al., 2018). Resultant energy-conserving behaviors—such as binge eating, huddling under blankets, oversleeping and reducing daily activities—align with depressive symptomatology. The scenario accordingly illustrates Barrett et al.’s (2016) proposal that the condition arises from the brain generating maladaptive predictive models that misallocate metabolic resources, a compelling position that still neglects several considerations.

First, trauma exposure is a well-established contributor to depressive trajectories and is particularly salient in pathways conferring elevated psychosis risk (Barlattani et al., 2023; Carpiniello & Pinna, 2023; de Miquel et al., 2022; Devries et al., 2013; Dworkin et al., 2017; Kendler et al., 2004; Kersting et al., 2011; Milner et al., 2013; Nanni et al., 2012; Paul & Moser, 2009; Z. Steel et al., 2009; Zisook & Shear, 2009). Yet the role of trauma in some cases of depression receives comparatively limited attention in allostatic predictive accounts. This is significant because trauma bears directly on normative judgments about whether depressive responses reflect dysfunction or context-sensitive adaptation.

Second, depressive presentations may sometimes just be energy-depleting physiological processes. For example, inflammation and sickness behavior associate with energy conservation (Dowlati et al., 2010; J. Li et al., 2018; Medzhitov, 2008; Miner & Kryger, 2017; National Institute on Aging, 2022). Older adults often show shifts in thermoregulatory preference toward warmer environments (Collins et al., 1981; Hwang et al., 2007; Rupp et al., 2015; Schellen et al., 2010). They also sleep more than younger people (de Miquel et al., 2022; Medzhitov, 2008; Paul & Moser, 2009). Though depression is heterogeneous in frequency and etiology among the elderly, research suggests high rates in certain cohorts, such as those in care facilities (Cormi et al., 2024; Dow et al., 2011; Grabowski et al., 2010; Gunawan & Huang, 2022; Hoover et al., 2010; Hu et al., 2022; Kramer et al., 2009; Melo et al., 2025; Seitz et al., 2010; Silva et al., 2024; Tang et al., 2022; F. Wang et al., 2021; Q. Wang et al., 2024; Zenebe et al., 2021). If these behavioral changes are based on predictive models, then the errors are minimal. Actions such as warmth-seeking are also adaptive and epistemically attuned to individuals’ physiological conditions and do not appear to involve significant prediction error.

A third consideration neglected by Barrett et al. (2016) is that temperamental dispositions that appear maladaptive in one context may be advantageous in another. This suggests that symptom expression may reflect environmental mismatch rather than intrinsic dysfunction.

To elaborate, bold fish have advantages when encountering novel food, unless it is in a baited trap, in which case their more wary counterparts fare better (Wilson et al., 1993). While one might object that an optimized fish would update predictive models according to the situation, this neglects that bold fish may not survive a first encounter with a trap, rendering revision moot. 

For a human example, consider how neuroticism correlates with creativity and intelligence in anxiety-prone individuals (Moutafi et al., 2006; Perkins et al., 2015), which may be advantageous in some contexts. Similarly, certain forms of autism can bestow advantages in employment fields requiring extreme attention to detail, systematizing, intense focus, bias avoidance and the ability to work in relatively solitary conditions (Armstrong, 2010; Crippen & Lindemann, 2024; Jurgens, 2023; Timimi & Leo, 2009; Uddin, 2022). While one should not conclude that autism never meets the threshold for being a disorder, assuming it always does risks imposing a somewhat arbitrary normative standard.

Barrett et al.’s (2016, p. 2) PP account of depression maintains that “anything which regulates (i.e., acts on) a system must contain an internal model of that system.” Yet the claim faces counterexamples: brainless slime mold communities regulate their internal milieu and solve complex environmental problems (Crippen, 2020), presumably without representations. Old work in artificial intelligence suggests the environment itself can serve as a more efficient model than internal representations (Feigenbaum, 1968), an idea echoed in various embodied accounts of mind (e.g., Dewey, 1896; J. J. Gibson, 1966; E. J. Gibson & Pick, 2000; Merleau-Ponty, 1945/1962; Varela et al., 1991).

Despite these considerations, PP accounts emphasize fine-grained neural regulation of bodily processes. In Barrett et al.’s (2016, p. 9) words, if “a senior colleague … approaches your office door, … your brain will predict your colleague’s arrival by constructing an embodied simulation,” thereby “redirecting blood flow to the legs from other organs that need it less,” computing “how much glucose is needed to stand up.” Aside from offering no supporting citations, Barrett et al.’s illustration raises further concerns.

First, it neglects peripheral regulation, such as contraction-induced vasodilatation whereby muscle use mechanically draws blood into active tissue (Kirby et al., 2007). Second, if bodily thresholds are so finely tuned that merely standing up requires temporary redirection of blood from vital organs, jogging could be life-threatening. Third, PP stresses efficiency, yet it is debatable how economical it is for the brain to micromanage such processes. Fourth, when PP theorists offer evidence, it is often circumstantial. For instance, R. Smith et al. (2020) infer that depressed individuals underestimate metabolic resources based on reduced synchrony in heartbeat tapping tasks. Whether this supports a global misestimation of metabolic capacity remains uncertain. Alternative interpretations, such as attentional disengagement from low-salience tasks among depressed individuals, are plausible.

At the same time, Barrett et al. (2016) are correct that depression can exert deleterious physiological effects. Inflammation, while adaptive in acute healing contexts, can damage surrounding tissue when prolonged; similarly, depression has been associated with structural and functional neural changes. One causal pathway is that depressive rumination involves sustained activation of the ventrolateral prefrontal cortex, potentially contributing to neural wear under chronic conditions (Andrews & Thomson, 2009). This may relate to ventrolateral prefrontal dysfunction linked to heightened expectations of negative social evaluation (Guyer et al., 2008; A. R. Smith et al., 2018), a pattern also emphasized in PP accounts.

These observations, however, do not decisively support the claim that depression constitutes an epistemic breakdown, or what PP accounts interpret as persistent gaps between expectations and available information (Barrett et al., 2016; Kube et al., 2020b; R. Smith et al., 2021). Instead, depression may sometimes follow from accurately recognizing unmet aspirations (Rottenberg, 2014), which does not entail predictive failures in the PP sense. On other occasions, it may reflect harm reduction, for example, because prospectively anticipating the recurrence of past trauma can lead to preventive measures. The result is prediction errors, but adaptive ones, insofar as they arise from expecting harm that does not materialize.

While sustained mismatch between top-down anticipation and bottom-up events can reflect precautionary calibration rather than epistemic failure, this does not make the overall situation desirable. Rumination is unpleasant. As stated, it also links to neuronal degradation and associated cognitive impairment (Andrews & Thomson, 2009; Guyer et al., 2008). But the outcome is analogous to muscle damage sustained while fleeing danger: intense contemplation and retreat may be advisable, even if occurring under circumstances most would wish to avoid, and even if downstream injuries warrant medical care (Crippen, 2025d, 2025a).

## 6. Prediction Error and Threat Avoidance in PTSD

PTSD can be profoundly impairing. Without minimizing the burden, which may become maladaptive in safe environments, this section reviews how PTSD-related vigilance may serve a protective function, increasing sensitivity to recurrent but unpredictable threats, supporting self-preservation (Cantor, 2005; Christopher, 2004; Crippen, 2025a; Nesse, 2019; N. A. Smith et al., 2019). To the extent that this mitigates danger, a discrepancy emerges between anticipated harms and comparatively benign outcomes. This observation complicates PP formulations that equate PTSD pathology with prediction errors (Linson & Friston, 2019; Lyndon & Corlett, 2020; Pierce & Black, 2024; Putica & Agathos, 2024; Kube et al., 2020a). The same scenario, as will be seen, raises questions about accounts that link these prediction errors to delusions and forms of psychosis (Linson & Friston, 2019; Lyndon & Corlett, 2020).

To begin with, anxiety is a recognized marker of PTSD (Cantor, 2005; L. L. Chao, 2016; Pigeon & Carr, 2016). Yet it also has epistemic significance (Miceli & Castelfranchi, 2005; Vazard, 2018), and moderate levels can improve task performance (Barlow, 2001; Eysenck & Calvo, 1992; Eysenck et al., 2007; Yerkes & Dodson, 1908). Because anxiety involves sustained anticipatory tension, it organizes experience into extended patterns of concern (Borkovec et al., 1998), fostering forward-looking counterfactual modeling (“what-if” cognizing) (Vazard, 2018). As with fear, anxiety accordingly heightens sensitivity to potential harms. However, whereas fear typically targets an identifiable object or event, anxiety is often more diffuse, arising in response to ambiguous or unspecified threats (Davis et al., 2010).

Corticotropin-releasing hormone (CRH) regulates arousal and wakefulness (Davis et al., 2010; Raglan et al., 2017). Although it serves non-stress functions as well, prolonged increases in its activity can sustain anxiety and heighten responsiveness to threat cues (Raglan et al., 2017). Accordingly, elevated CRH in PTSD may be deemed “predictive” insofar as it enhances vigilance and sensitivity to potential danger (Crippen, 2025a). On temporally extended scales, anxiety may likewise modulate fear sensitivity and bias information processing toward risk (see Kurth, 2018).

Within a PP framework, such bias can be operationalized as increased precision-weighting of threat-related priors, such that ambiguous stimuli are preferentially interpreted through danger-expectant generative models (A. Clark, 2013; Friston et al., 2017). Sustained arousal can further amplify interoceptive prediction errors and stabilize higher-level threat anticipation, reinforcing risk-sensitive behaviors (Hesp et al., 2020; Seth & Friston, 2016). Elevated anxiety is a core feature of PTSD, which may develop following sexual violence, combat exposure or severe accidents (APA, 2022). Neurobiological differences are frequently observed in affected individuals, though it remains unclear whether these reflect trauma-induced alterations, pre-existing susceptibility or their interaction (Hendler & Admon, 2016). Increased CRH signaling has been linked to PTSD pathophysiology and may contribute to altered arousal regulation and heightened vigilance (Bremner et al., 1997; Dunlop et al., 2014).

PP accounts of psychosis propose that aberrant precision-weighting—through overly precise priors or dysregulated prediction-error signaling—can yield hallucinations and persecutory ideation when expectations of harm dominate perceptual inference (Adams et al., 2013b; Corlett et al., 2016; Sterzer et al., 2018). In PTSD, sustained anticipation of adversity may, under certain conditions, increase vulnerability to paranoia by entrenching danger-biased models that reshape explore–exploit dynamics during chronic stress (Kube et al., 2020a; Linson & Friston, 2019). The hypothesis aligns with broader trauma-to-psychosis pathway models that identify post-traumatic intrusions, negative beliefs and threat appraisal as routes to paranoid interpretations (Hardy, 2017; Wilkinson & Bell, 2016).

Non-PP researchers also characterize PTSD accounts as involving a persistent and distorted sense of peril (Ehlers & Clark, 2000; Shalev et al., 2017), and infrequent but consequential threats may exacerbate worries, impeding extinction when safety returns (Crippen & Schulkin, 2020; Stillman & Aupperle, 2016). Still, context matters. What appears paranoid in ordinary circumstances—say, believing that world leaders want you dead—may accurately reflect the plight of soldiers, as expressed by the protagonist in the WWII novel *Catch-22* (Heller, 1961). Along these lines, neurobiological work shows that defensive responses often scale with the perceived proximity and severity of danger (Davis et al., 2010; Mobbs et al., 2007). Evolutionary models further suggest that, under asymmetric cost conditions, selection favors false-positive biases when the cost of missing a real threat is high (Haselton & Buss, 2000; Nesse, 2005). The resulting arousal is physiological as well as cognitive, as worry forecasts or represents future peril (Miceli & Castelfranchi, 2005; Crippen, 2018, 2021a; Miloyan et al., 2016). By definition, the menace has not been actualized, yet it need not be fictive if it materializes or there is a reasonable expectation that it will.

Yet there is a sense in which certain strong PP proponents would deny the last claim even before considering PTSD. This is because some—but not all—explicitly describe perception as a kind of hallucination—albeit one that functions well when it corresponds to practical environmental demands within a certain tolerance of error (Barrett et al., 2016; Hohwy, 2013; Ongaro & Kaptchuk, 2019; Wilkinson et al., 2017). The idea is that agents perceive predictive models constructed inside the brain—its guesses or hypotheses about the world (Barrett et al., 2016; Friston, 2010; Hohwy, 2013; Ongaro & Kaptchuk, 2019; Wilkinson et al., 2017; A. Clark, 2016). Less controversially, one might speak of hallucinated absences in relation to model-based omissions that blind observers to environmental features that are more distracting than useful. PP advocates often attribute mental illness to failures to do this, not just with PTSD, but other conditions like ADHD.

The student with ADHD mentioned earlier, whose expectation of a quiet room is disrupted by a tennis game many floors below, may be contrasted with another case raised at the outset: Ariaal nomads. These herders traditionally move through dynamic landscapes, not static settings, and rely on subtle sounds (O. Chao et al., 2025). For the nomads, a pathology judgment is misplaced, and for the student it is circumstantial and norm-dependent. After all, ADHD traits can bestow advantages if one wanders restlessly between topics while noting details, resulting in high-quality interdisciplinary work. Moreover, the low-margin-of-error prior may result from heightened sensitivity rather than causing that outcome, and may advantageously lead the person to seek a more suitable space.

For PTSD, the literature lists many associated deficits, including diminished neural suppression of rapidly repeated auditory stimuli (Villarreal & Hunter, 2016). PP frameworks interpret this phenomenon in terms of hyperprecise (narrow) thresholds for sensory prediction errors (A. Clark, 2016; den Ouden et al., 2012; Feldman & Friston, 2010; Friston, 2010; Hohwy, 2013; Pellicano & Burr, 2012; Van de Cruys et al., 2014). But for people dealing with ongoing violence—whether war or sexual abuse—faint metallic clicks or barely perceptible floor creaking might justifiably violate an “all-safe” predictive model. While heightened acuity here seems appropriate, PP’s “hyper-“ prefix insinuates pathologically, if not delusionally, precise priors. The framing inserts a normative judgment about how brains should function—about the appropriate neurocomputational balance between tolerating more false alarms versus missing fewer dangerous cues.

This reconstruction exposes a tension within PP accounts. The question is under what environmental conditions sustained anticipatory models should be considered pathological rather than strategically calibrated. Instead of reflecting overly narrow thresholds for sensory prediction errors, the lack of neural suppression may reflect greater gain assigned to low-salience but important cues. In high-risk environments, ordinarily negligible acoustic changes can signal imminent danger, and trauma survivors have reported heightened vigilance and threat sensitivity (Crippen, 2025d). Intermittently occurring threats are difficult to definitively rule out. Because sexual assault survivors are frequently revictimized (Barnes et al., 2009; Sorenson et al., 1991; H. E. Walker et al., 2019), trauma-exposed individuals may reasonably retain defensive priors even after leaving overtly dangerous settings. This generalized persistence can manifest as sustained anxiety and rapid threat reactivity, which may—yet need not—be maladaptive or delusional.

## 7. Normative Framings of Trauma in Predictive Processing

The evidence analyzed so far suggests that PP framings overstate the maladaptive aspects of psychological conditions, though the criticism also applies to other perspectives on mental health. PP accounts additionally include unacknowledged normative insertions—not only in what they label as maladaptive, but in somewhat arbitrary adjudications of either too much or too little precision as epistemically distorting. Furthermore, PP interpretations can inadequately recognize how differing life circumstances should shape such determinations. This section elaborates on these points in the context of PTSD.

On the one hand, some PP accounts of PTSD acknowledge aspects of what has been said so far. A case in point is Wilkinson et al. (2017), who discuss hierarchically structured hypotheses centered on a prior life-threatening event. Since the event’s existential salience is high, the brain assigns high precision even to low-probability stimuli signaling potential recurrences, so that a wide range of cues—some not indicative of danger—elicit traumatic re-experiencing. Still, flashbacks are usually not confused with present reality, as higher-level contextual beliefs about time and location remain operative.

On the other hand, alternative explanations follow from the fact that schizophrenia and related psychotic-spectrum conditions can co-occur with PTSD (Fleming & Martin, 2016). Just as radar can be rewired to produce atypical readings without predictive models or their corruption, the nervous system may generate anomalous signals without distorting priors. It is also unclear how much PP’s complex theoretical machinery really illuminates the familiar finding that trauma reminders trigger vivid recollection, and Wilkinson et al. (2017) provide little supporting evidence from memory or neuroscience research.

To elaborate, hallucinations are rare in PTSD unless sufferers meet diagnostic criteria for schizophrenia (C. Steel, 2015). Schizophrenia associates with altered neurotransmitter activity, neural connectivity and synaptic signaling, plus inflammatory, oxidative and immune stress, including imbalances in gut microbes (Ding, 2020; Galińska-Skok & Waszkiewicz, 2022; Luvsannyam et al., 2022; Rawani et al., 2024; Severance et al., 2016; Sierakowska et al., 2025; Thaker, 2008; Tomasik et al., 2016). Evidence suggests interactions between genetic, neurodevelopmental and environmental influences (Stöber et al., 2009). In short, PTSD-related hallucinations—if linked to schizophrenia—may parallel the unusually wired radar rather than automatically indicating predictive models gone awry.

This critique does not diminish the clinical seriousness of PTSD. For example, elevated amygdalar firing and hippocampal shrinkage often accompany the condition (Bukhbinder & Schulz, 2016; L. L. Chao, 2016; Hendler & Admon, 2016; Karl et al., 2006; Stillman & Aupperle, 2016). Though distinguishing predisposing vulnerability from trauma-induced change remains challenging, prolonged stress hormone elevation links to glucocorticoid neurotoxicity and hippocampal atrophy (Lau et al., 2016).

That said, glucocorticoids also contribute to survival mobilization and heightened vigilance via adrenergic interactions (Sherin & Nemeroff, 2011). To label such responses as necessarily “maladaptive” is misleadingly akin to judging a soldier’s instinctive flight from a rolling barrage as pathological simply because it results in a sprained ankle. In both cases, downstream injuries—whether stress-related neural atrophy or musculoskeletal strain—may warrant treatment. Yet this does not render the precipitating actions inherently disordered.

In sum, the symptomatology of PTSD includes emotional numbing, detachment, avoidance, sleep problems, concentration difficulties, impaired inhibitory control and anxious hypervigilance (Depue et al., 2014; Jovanovic & Norrholm, 2011; King et al., 1998; Norman et al., 2007). These symptoms are frequently framed in normatively loaded terms. Hypervigilance implies excessive levels. But it may instead reflect reasonable caution; anxiety, avoidance and sustained wakefulness can be epistemically appropriate insofar as they support “what if” simulations of real but intermittent threats. Likewise, impaired inhibitory control may amount to increased reactivity when slow reflection is costly. Emotional numbing and detachment may be functional, since fear can be paralyzing while extreme sympathy for an attacker or even a neighbor’s children is usually unproductive when under assault. Finally, concentration difficulties might reflect diverted attention in contexts such as warzones, where a soldier usually misallocates cognitive resources by tracking her heartbeat or pondering the literature of Alejandra Pizarnik, Han Kang or Naguib Mahfouz.

Neuroimaging findings broadly coincide with this interpretation. Individuals with PTSD often exhibit reduced activity in anterior cingulate and prefrontal regions relative to others (Shin et al., 2001). These regions are implicated in higher-order attentional regulation, impulse control and deliberative decision-making. Diminished activation may therefore indicate a shift away from reflective, strategic processing toward faster, more automatic responding (Kurth, 2018). This is particularly when accompanied by heightened amygdala reactivity that biases the system toward reflexive emotional responses. Contrary to standard assumptions, such profiles may confer practical advantages under conditions of extreme and unpredictable danger. In active combat zones, for example, it would be maladaptive—not prudent—for soldiers to pause and deliberate over whether a sudden explosion is fireworks rather than an attack (Crippen, 2025d, 2025a).

It is worth reiterating that none of this denies that trauma can result in pathological outcomes. A war veteran who develops PTSD may experience a condition that unsettles her sense of reality and alters her relationships with others years after she has left the combat theater. Perhaps here most can agree that PTSD markers are maladaptive.

But the situation may be very different for a sexual abuse survivor, even after an extended period of safety since the risk of revictimization is significant (Barnes et al., 2009; Sorenson et al., 1991; H. E. Walker et al., 2019). For someone who feels she could not cope with the idea of another assault, it may be practically sensible to take even unlikely threats seriously to lower overall risk over time. Her world may come to feel saturated with threat, reshaping expectations and heightening vigilance.

Such an orientation need not be interpreted as delusional; rather, it may reflect context-sensitive calibration to lived danger—an affective stance aimed at guarding against unwarranted assumptions of safety.

PTSD may sometimes involve re-exposure—for example, abusive relationships (Fereidooni et al., 2023). Clinical intervention may be appropriate. Still, renewed exposure is often difficult to avoid because men statistically constitute the primary source of interpersonal danger across many environments, particularly for women (Crippen, 2025e). Although this does not imply that any given man poses danger, cumulative risk matters. Hence, the normative threshold separating adaptive calibration from pathological overcalibration cannot be determined solely from neurocomputational parameters such as precision weighting; it depends on ecological risk structure and the asymmetric costs of error.

In PP terms, greater tolerance for false positives may reduce severe harm when missed signals are costly. Although a less guarded stance might not culminate in revictimization, this can only be known retrospectively, and trauma survivors may reasonably regard certain statistical risks as unacceptable.

Accordingly, norm-infused evaluations warrant caution—for example, characterizing PTSD as involving “perception of threats where none exist” (Barrett et al., 2016, p. 9), or celebrating a psychopharmaceutical for reducing “avoidance of trauma-related activity by week 10” (Davidson, 2006, p. 35). Likewise, the claim that PTSD involves assigning “more negative and threatening interpretations than [events] warrant” (Wilkinson et al., 2017, p. 7) may insufficiently acknowledge the existential weight of objectively hazardous circumstances. Finally, restricting diagnosis to cases in which alarm responses are demonstrably false would implausibly exclude individuals living in active combat zones or abusive domestic settings from PTSD diagnosis, undermining the objection’s credibility.

## 8. Hallucinated Perception or Constructed Reality?

Proponents often trace PP to eighteenth- and nineteenth-century work by Bayes and Helmholtz (A. Clark, 2016; Fletcher & Frith, 2009; Friston, 2010; Hohwy, 2013; Pellicano & Burr, 2012; Rao & Ballard, 1999). Its intellectual lineage, however, extends further back to early Modern representational theories of mind. In contemporary computational psychiatry and neurobiological models of psychosis, these commitments are formalized in Bayesian probabilistic frameworks that explain perceptual disturbance, delusion formation and aberrant salience attribution. In this context, proponents frequently describe perception as “controlled hallucination” (Wilkinson et al., 2017, p. 4) and state that “your brain is wired for delusion” (Barrett, 2017a, p. 66). Less polemically, agents experience “the brain’s best guess” rather than the world itself (Ongaro & Kaptchuk, 2019, p. 1). On this view, the brain constructs “internal models of the body in the world,” tested against incoming sensory evidence (Barrett et al., 2016, p. 1).

PP authors often note that affectively primed expectations can shape perceptual decision-making—for instance, making individuals more likely to report seeing a gun when none is present (Fridman et al., 2019; Neemeh, 2020). In neurocomputational psychiatry, such findings are typically interpreted through shifts in precision-weighting or threat-related priors, frequently discussed in relation to psychosis-spectrum and trauma-related conditions.

Consider a study conducted after the 2013 Boston Marathon bombing, reporting that angry participants living in the metropolitan area were more likely to misperceive people holding neutral objects as armed (Wormwood et al., 2017). Stimuli appeared for only 500 milliseconds, while response time was unlimited. However, image resolution was low enough to be highly ambiguous even with extended viewing, undercutting claims about misperception.

Nonetheless, two authors of the just-cited study—Barrett and Wormwood—state in another article that “neural ‘guesses’ largely shape what you see, hear, and otherwise perceive” (Barrett & Wormwood, 2015, para. 5). Discussing the shooting of harmless citizens, Barrett elsewhere adds: “Human brains are built for this sort of delusion, through the same process that produces daydreams and imagination” (Barrett, 2017a, p. 236). This comes close to suggesting that psychosis-like processes are normal, though not always harmful.

There is a variant of Wormwood et al.’s (2017) paradigm that presents participants with non-ambiguous images but imposes strict time limits on identification (Baumann & DeSteno, 2010; Payne, 2001; Wormwood et al., 2015). Misattributions of guns again occur. But when participants later restated their answers outside the rapid-response window, error rates fell to near zero (Baumann & DeSteno, 2010).

The response pattern may therefore reflect transient inhibitory-control constraints under speeded conditions rather than predictive aberrations leading people to see what is not there. Comparable effects occur with neutral stimuli. For example, a common neuropsychological test requires participants to press a button for frequent target letters and withhold responses to rare non-targets. It thereby measuring impulse control instead of perceptual distortion (Beck et al., 1956; Bellani & Brambilla, 2011; Dougherty et al., 1999, 2003; Riccio et al., 2002). This last point is obvious to those who have undergone the test.

The cited studies on threat assessment therefore do not show that top-down priors directly rewrite perceptual content. Rather, they indicate that negatively valenced affective states modulate downstream response selection, decision thresholds and action tendencies, whether or not predictive models drive them.

Prior beliefs and emotions may, however, still alter perception in ways that do not involve predictive processing. With depression—to repeat an example—the world is not merely seen (or hallucinated) as more onerous; it can become so because fatigue, inflammation or psychomotor slowing reduce a sufferer’s capacity to act, making goals objectively harder to attain (Bennabi et al., 2013; Dantzer et al., 2008; Felger & Miller, 2017; Goldsmith et al., 2016; Miller & Raison, 2016). In this sense, depression can be understood as a bodily enactment of a more effortful environment (Ratcliffe, 2015). Avoidance of social gatherings may thin networks of support, just as persistent expectations of failure may be partly realized when reduced motivation leads individuals to avoid demanding tasks (Foa & Kozak, 1986; Orth & Wieland, 2006; Treadway et al., 2009; Turner et al., 2022; Yang et al., 2014).

PTSD has comparable dynamics. The condition associates with chronic irritability or defensive aggression (Orth & Wieland, 2006; Taft et al., 2016; Van Voorhees et al., 2016; Wilk et al., 2015; N. Zhan et al., 2024). This, in turn, can provoke hostility in others, generating a social environment that both reflects and amplifies symptoms. Such feedback loops may stabilize threat-related expectations or interpretations overlapping with mechanisms often discussed in psychosis research (De Rossi & Georgiades, 2022).

Thus, altered affect is not merely an internal disturbance but a way of engaging the world that actively shapes the field of possibilities available to the agent. At one level, PP addresses this through active inference, whereby behavior brings about environmental changes and sensory inputs that align with an organism’s predictive models (Seth, 2013; Seth & Friston, 2016). On this view, organisms do not simply “imagine” a world—they help structure conditions that confirm their expectations.

Even so, a broadly hallucinatory emphasis continues to run through many PP formulations, including those stressing active inference, such as Barrett et al.’s (2016) version. In fact, PP can have a neurocomputational flavor that mirrors the movie *The Matrix*, where characters mistake an AI-governed dream world for reality. This comparison is based not only on the PP claim that perception is a “controlled hallucination” (Barrett et al., 2016; Ongaro & Kaptchuk, 2019; Wilkinson et al., 2017). It also rests on PP assertions that mental representations can directly generate bodily harm (Barrett et al., 2016; Ranum et al., 2022; Sharp et al., 2021), a premise central to *The Matrix*.

Delusional paranoia provides a straightforward case in which brain-generated representations appear to produce bodily harm, since hostile suspicion toward others may provoke retaliatory violence. Barrett et al. (2016), along with other PP theorists (Ranum et al., 2022; Sharp et al., 2021), also advance an active inference account according to which the brain deploys energy-dysregulating priors. This generates internal sensory inputs (fatigue, agitation, etc.) that correspond to the brain’s predictive model.

To elaborate, a subset of patients with depression and comorbid conditions such as chronic fatigue syndrome or fibromyalgia exhibit low cortisol levels (Penninx et al., 2007; Tops et al., 2008). A PP framing might interpret this as the result of the brain persistently anticipating sleep—for example, through high precision thresholds assigning significant weight to slight energy depletion. This, in turn, could reduce cortisol, which typically decreases as one becomes drowsy (Juliana et al., 2025; O’Byrne et al., 2021; Sukor et al., 2025). As a result, feelings of exhaustion and related interoceptive signals would align with—and be reinforced by—the predictive model that simultaneously elicits them. The upshot, from this standpoint, is that neurally instantiated expectations can give rise to genuine somatic pathology (Barrett et al., 2016; Ranum et al., 2022; Sharp et al., 2021).

Once more, however, causality may run the other way, as inflammation, sickness, fatigue and immune activity shape brain function (Dantzer et al., 2008; C.-H. Lee & Giuliani, 2019). This was already noted in earlier discussed work on gastrointestinal- and starvation-linked depression (Andrews et al., 2020). These cases also bear on atypical depression, where irritable bowel syndrome is frequently comorbid (Fries et al., 2005; Tops et al., 2008). The condition itself is exhausting, and cortisol may decline partly to promote rest. Perception may accordingly undergo alterations, with fatigue shown to make distances appear greater (Bhalla & Proffitt, 1999; Proffitt, 2006; Proffitt et al., 2003; Schnall et al., 2010; Stefanucci et al., 2008; Witt, 2011; Zadra et al., 2010). But these changes do not vindicate PP claims that the brain is wired for delusion or controlled hallucination. Instead, agents may be accurately calibrating to the reality that environments and tasks are objectively harder to navigate (Crippen, 2025d, 2025a).

Comparable examples abound. Sad individuals show reduced exploratory impulses and a preference for enclosed spaces (Mealey & Theis, 1995), which aligns with their vulnerability. Anxious participants in wall-climbing tasks reach cautiously and less far, such that intervals between handholds appear larger (Graydon et al., 2012; Pijpers et al., 2006). People exhausted by financial strain likewise register stairs as steeper, coinciding with the fact that they are harder to climb (Liu et al., 2018). In such cases, there appears to be little prediction error, since perception reflects bodily attunement to environmental challenges.

According to this action-based standpoint, even depressive torpor, binge eating and inflammatory responses can be adaptive. For example, if winter in tenth-century Iceland prevented significant outdoor work, an inflammatory slowdown might increase sleep, preserving calories, especially when combined with heavy eating, building fat and aiding thermoregulation (see Cannon & Nedergaard, 2004; Crippen, 2025a, 2025b; Hart, 1988; Sherman et al., 1991; Wells, 2006).

In predictive terms, there would be little discrepancy between top-down expectations and bottom-up inputs for seasonally deflated tenth-century Icelanders. Reduced activity would fit winter ecologies and interoceptive signals of lowered vitality, consistent with accounts of interoceptive predictive regulation.

Moreover, because demanding productivity standards were unlikely to structure daily life in that context, lethargy might not be experienced as pathological. The critique nonetheless partly vindicates PP accounts: what renders seasonal low mood a “disorder” today may be the widening gap between normative expectations of sustained output and winter-related reductions in capacity.

## 9. Predictive Processing and Schizophrenia

Schizophrenia may comprise a cluster of different brain conditions with similar symptom profiles (Arnedo et al., 2015a, 2015b; Insel, 2010). Regardless of this underlying heterogeneity, the diagnostic category is often treated as a paradigmatic case of psychosis because of its association with hallucinations and paranoid delusions. Since general PP accounts of these phenomena have already been reviewed, this section focuses on a more specific proposal: that schizophrenia arises from impaired prediction of the detectable consequences of one’s own actions (Ford & Mathalon, 2012; Friston, 2012; Griffin & Fletcher, 2017; Okimura et al., 2023; Rossetti et al., 2025). On this view, if individuals have difficulty anticipating their own inner speech or behavior as self-generated, they may experience auditory hallucinations or perceive themselves as being taken over by outside forces (Rossetti et al., 2024).

There are, however, potential problems in this interpretation, aside from the fact that brain imaging does not directly reveal predictive models, representation and computational structures (Bechtel & Abrahamsen, 2005; Bowers & Davis, 2012; Henson, 2005; Hodson, 2024; Miłkowski & Litwin, 2022; Poldrack, 2006, 2011; Poldrack & Wagner, 2004; Qela et al., 2025). One issue is that the outlined PP accounts rely on Westernized norms about self and agency, even though both vary in complex ways across and within cultures (Bart et al., 2019; Colnaghi et al., 2026; Crippen, 2021b, 2023, 2024, 2025c, 2025f, 2025g; de Oliveira & Nisbett, 2017a, 2017b; Dudgeon et al., 2022; Ge, 2026; Han et al., 2022; Ho, 1995; Hong et al., 1997; Hornsey et al., 2019; Ji et al., 2000; Jojola, 2003; Joo et al., 2022; Kim, 2021; Kirmayer, 2004; Kitayama & Salvador, 2024; Markus & Kitayama, 1991; Mbiti, 1970; Raj et al., 2018; Rivera Andía, 2018; Roland, 1988; Salvador et al., 2026; Sastry & Ross, 1998; Tafarodi et al., 2004; Vignoles et al., 2016; G. Wang et al., 2012; Y.-M. Wang et al., 2025; Whitt et al., 2001; Yamaguchi et al., 2005; Zafar et al., 2025).

For example, one study of 46,503 individuals across 33 countries found that low agency predicts mental illness in Western contexts but not in East Asia (Sastry & Ross, 1998). There, people report greater social interdependence, aligning with regional values that emphasize adapting oneself to specific situations to promote harmony (Han et al., 2022; Ho, 1995; Markus & Kitayama, 1991). By contrast, Americans often idealize agency and report higher levels of it (Crippen, 2025a; Sastry & Ross, 1998).

Now, assuming basic PP assertions about the self and schizophrenia are correct, one might expect prevalence to be higher in regions characterized by greater interdependence and reduced emphasis on individual agency. Although some evidence suggests this may be the case in Japan, most Asian countries report lower schizophrenia prevalence than the United States (Our World in Data, 2023; World Population Review, 2026b; Z. Zhan et al., 2025).

Furthermore, a 2026 systematic review and meta-analysis of 60 articles covering 20,910,871 individuals shows very low rates in Japan, China, South Korea and India—together about four times lower than the US (S. Zhang et al., 2026). Across these datasets, sub-Saharan rates are also modest (Our World in Data, 2023; World Population Review, 2026b; Z. Zhan et al., 2025; S. Zhang et al., 2026).

These observations do not necessarily preclude PP explanations; yet they do raise a question about whether PP framings apply locally rather than globally.

The rest of this section and the next address this issue, first by providing more detail on how PP proponents link schizophrenia to aberrant models of self and agency; second, reviewing cultural variation in self and agency; and third, considering potentially confounding variables, such as family socioeconomic status at birth, which correlates inversely with both agency and schizophrenia (Callan et al., 2024; Corcoran et al., 2009; Esposito, 2003; K.-J. Huang, 2026; Landau, 1995; Werner et al., 2007).

PP theorists argue that sense of agency depends on individuals correctly predicting what their actions will feel and look like. If the brain does not appropriately anticipate perceptual inputs resulting from its own decisions, then self-generated stimuli—whether internal or environmental—are not properly “tagged” as one’s own (Ford & Mathalon, 2012; Fletcher & Frith, 2009; Friston, 2012; Griffin & Fletcher, 2017; Sterzer et al., 2018).

Accordingly, PP accounts of schizophrenia treat disturbances of agency and sense of self as failures of predictive inference, particularly in how the brain anticipates the consequences of its own actions (Fletcher & Frith, 2009; K.-J. Huang, 2026; Griffin & Fletcher, 2017; Sterzer et al., 2018). As a result, internally generated signals (like inner speech) can be experienced as external (e.g., hearing voices), or one’s own movements may feel controlled by something else (Ford & Mathalon, 2012). PP theorists also argue that aberrant prediction errors can drive maladaptive belief updating, helping stabilize delusional interpretations of anomalous experiences (Adams et al., 2013b; Corlett et al., 2016).

Computational PP work proposes that part of the problem may be timing: if predictive signals are delayed, the system cannot properly match actions with their outcomes (Arnedo et al., 2015b; Ford & Mathalon, 2012; Friston, 2012)). This can produce both reduced and exaggerated senses of control: patients may feel their actions are controlled by others or, conversely, that they caused unrelated events (Okimura et al., 2023; Rossetti et al., 2025). The scenario also fits broader PP accounts of hallucination emphasizing overweighting of priors in perception (Corlett et al., 2019; Powers et al., 2017).

Several PP models also describe schizophrenia as a disruption of the brain’s self-model (Fletcher & Frith, 2009; Griffin & Fletcher, 2017; L. Li & Li, 2025; Sterzer et al., 2018). When precision-weighting and sensorimotor integration break down, the boundary between self and world becomes unstable, leading to forms of disembodiment (L. Li & Li, 2025). At the same time, aberrant salience—driven by misweighted prediction errors—makes irrelevant internal or external signals seem unusually important (Adams et al., 2013b; Corlett et al., 2016; Pugliese et al., 2022; Sterzer et al., 2018). This distorts judgments about what caused an event, including whether it was self-generated, contributing not only to delusions and hallucinations but also to disturbances of agency (e.g., thought insertion, delusions of control) (Pugliese et al., 2022).

Rossetti et al. (2024) elaborate in a review. For people with schizophrenia, the authors conjecture that a weakened basic “this is my action” signal generates feelings of being manipulated by outside forces; or else, exaggerated expectations of control may inflate agency, producing a false sense of omnipotence. The account fits PP framings emphasizing imprecise sensorimotor predictions and overly strong higher-level priors (A. Clark, 2013; Gordon et al., 2017; Shao & Beck, 2024). Rossetti’s team maintains that weakened low-level self-prediction is the core problem, with over-attribution arising secondarily.

In a follow-up experimental study, Rossetti et al.’s (2025) team empirically examined this proposal. They did so by testing intentional binding effects—defined as the subjective temporal compression between a voluntary action and its sensory consequence, with shorter perceived intervals taken to indicate higher agency. Patients with schizophrenia showed reduced or absent binding, which Rossetti et al. interpret as a weakened link between action and outcome, thus diminished agency.

That said, recent work on intentional binding presents complications that Rossetti et al. (2024, 2025) do not consider. For instance, sexual arousal and depressive traits diminish the effect (Render & Jansen, 2021; Scott et al., 2022; Vogel et al., 2024). The latter may reflect reduced control due to exhaustion, without necessarily implicating an aberrant predictive model. Depression is comorbid with schizophrenia (Etchecopar-Etchart et al., 2021; W. Li et al., 2020), and Rossetti et al.’s (2025) results could therefore be partly an artifact of this, without substantiating PP interpretations.

So, one complication is that a person—due to exhaustion, say—could experience lower agency and less time compression without a distorted predictive model, meaning Rossetti et al. may infer too much from their data. As a general point, it is worth adding that overreaches of PP are not just empirical but conceptual as well. One example is reliance on rewordings. Sense of self morphs into predictive self-models (Apps & Tsakiris, 2014; Okimura et al., 2023). Object recognition becomes perception through prior models (Aitchison & Lengyel, 2017; A. Clark, 2016, 2013; Hohwy, 2013; de Lange et al., 2018; Pellicano & Burr, 2012; Rao & Ballard, 1999). Neural activity gets recast as predictive signaling (Horga et al., 2014; Okimura et al., 2023).

A second issue is that Rossetti et al.’s sample comes from an Italian hospital, yet intentional binding and agency vary across cultures. One experiment found that people from geographically Western nations showed stronger intentional binding for pleasant than for irritating outcomes, a difference absent for participants from places like China, the Middle East and South Asia (Barlas & Obhi, 2014). Among Chinese, Tibetan and Uyghur participants, intentional binding increased when individuals were primed with culturally congruent cues, such as images of traditional clothing, suggesting group identity can enhance volitional experience (M. Li et al., 2019). Austrian participants reported greater control when stimuli followed their actions quickly, whereas timing had little effect on Mongolian participants (Bart et al., 2019).

Thus, while PP theorists link schizophrenia to aberrant models of self and agency, this begs the question: aberrant from whose perspective? Also, if self and agency vary across cultures, should one expect corresponding shifts in schizophrenia rates? The next section considers these matters.

## 10. Predictive Processing, Culture and Psychosis

Cultural claims overgeneralize by neglecting internal heterogeneity and social evolution (Ess, 2014). Still, regional differences in agency and self-construal are well documented. These patterns pertain to PP accounts of schizophrenia discussed in the last section.

Rossetti et al. (2025), as outlined earlier, propose aberrant predictive models of agency as contributing to schizophrenia. However, agency is complex. It can correlate with independent self-concepts and inversely with interdependent construals (Sastry & Ross, 1998). Yet independent and interdependent self-understandings can also associate positively (Konakawa et al., 2026; Raj et al., 2018), and collectivism may support individual agency (Crippen, 2025c; Ferrari, 2022).

Additionally, there are different kinds of agency. Participants across cultures tend to prefer primary over secondary agency—that is, exerting control rather than adapting to situational constraints. Nonetheless, secondary agency gets stronger endorsement from Japanese and Japanese Canadians, as compared to European Canadians (Han et al., 2022).

These results already put pressure on Rossetti et al.’s (2025) position. As noted, low agency poorly predicts mental illness in Japan (Sastry & Ross, 1998), with recent work indicating low schizophrenia rates there (S. Zhang et al., 2026). Additionally, agency—whether measured through intentional binding or other paradigms—varies across cultures (Barlas & Obhi, 2014; Bart et al., 2019; Han et al., 2022; Hornsey et al., 2019; Ji et al., 2000; M. Li et al., 2019; Sastry & Ross, 1998; Yamaguchi et al., 2005). Rossetti’s team therefore may overgeneralize from their small Italian sample.

In cultural psychology, either–or binaries often fail, and as noted earlier, interdependence—though more common outside the West—can exist alongside high independence in the same person (Konakawa et al., 2026). Ubuntu perspectives in Africa, for example, view the self as highly relational, with certain privacy boundaries defined at the community rather than individual level—similar to how Western families share some matters internally but not externally (Crippen, 2021b). However, rural South African women may still value personal autonomy while pursuing it through peer involvement and social relationships (Ferrari, 2022).

Also challenging simple binaries, Latin Americans often combine interdependence with high levels of individual self-expression and assertiveness (de Oliveira & Nisbett, 2017a; Vignoles et al., 2016). Psychological well-being in these contexts may not map straightforwardly onto agency. It may instead relate to how successfully individuals translate agency and socioeconomic resources into culturally valued ways of living (Dressler et al., 2024).

In Amerindian historical contexts, the self has often extended beyond the human to include animals, spirits and landscapes as persons within familiar genealogies (Dudgeon et al., 2022; Ingold, 2011; Jojola, 2003; Rivera Andía, 2018; Viveiros de Castro, 1998, 2004, 2007, 2014, 2016; Whitt et al., 2001). Nonetheless, personal autonomy persists (Mann, 2005), often scaffolded by nomadic histories (G. Lee et al., 2025) and flexible social structures (Mann, 2005). For some of Australia’s Aboriginal peoples, the self is embedded in the land as an agentive meshwork of ancestors, places and non-human beings (Harvey, 2005). “Sentient ancestors transform themselves into ‘objects’ such as hills and [carved] boards, which then become the fixed points by which Aboriginal people individuate themselves as mature subjects” (Harvey, 2005, p. 73).

Work on South and East Asia suggests the self is often interdependent, yet contextual signals can shift this, with Eastern or Western cues priming people, respectively (Hong et al., 1997; Roland, 1988; Y.-M. Wang et al., 2025). These effects are stronger among younger participants (Roland, 1988; Y.-M. Wang et al., 2025). Other data indicate that Americans skew toward independence and Japanese toward interdependence; these categories correlate inversely in those cultures but are not dichotomous, as independence and interdependence correlate positively for Chinese participants (Konakawa et al., 2026).

Further challenging the individual–collective binary, Korean and Chinese students value group work more than Westerners while appreciating it for exposure to diverse opinions; they likewise prefer class discussion, likely for similar reasons (Crippen, 2025c). Substantial within-region variation also occurs. For example, interdependence and independence are higher, respectively, in historical rice versus wheat farming regions in China (Talhelm et al., 2014). Rice cultivation requires greater coordination than wheat, fostering more interdependent self-construals (Talhelm & Dong, 2024; Talhelm et al., 2014).

Evidence suggests culturally based cognitive, perceptual and neural variations (Boduroglu et al., 2009; Chiu, 1972; Cui et al., 2015; Ge, 2026; Hedden et al., 2008; Ishii et al., 2003; Kitayama & Ishii, 2002; Kitayama et al., 2003; Masuda & Nisbett, 2001; Šašinková et al., 2023; C. Wang et al., 2014; Waxman et al., 2016; Yee, 2020; Yu et al., 2021; Zhu et al., 2007). Experiments show that Westerners tend to focus on foreground objects. East Asian attention is more holistically distributed across contexts (Boduroglu et al., 2009; Masuda & Nisbett, 2001). Greater gray matter volume in regions supporting this type of processing associates with interdependent self-construals (Yu et al., 2021), consistent with a greater sense of extending into the world. However, while the authors take this to show that “culture goes deep under the skin and is eventually ‘embrained’” (Yu et al., 2021, p. 8)—a view PP theorists might take as supporting their concept of priors—the premise has a flipside: environments scaffold brains, psychic activity and perhaps predictive models as well.

Even apart from mental illness, these empirical observations should interest PP advocates, since—if the theory holds—the findings point to culturally inflected priors, including ones relevant to schizophrenia. Explaining the point takes several steps.

First, the idea of an environmentally scaffolded brain is reinforced by neuroimaging of the medial prefrontal cortex (MPFC) and anterior cingulate cortex (ACC), both associated with self-processing. For Chinese participants, who are typically more interrelationally disposed than Westerners, thinking about either their mothers or themselves activates these regions. In contrast, Americans show this pattern only when reflecting on themselves (G. Wang et al., 2012; Zhu et al., 2007).

Second, schizophrenia associates with altered MPFC and ACC functioning, suggesting that symptomatic voice hallucinations may arise from individuals generating dialogue without recognizing it as self-produced (Cui et al., 2015). Here, culturally inflected selfhood appears to moderate the condition. Luhrmann et al. (2015) found that Americans often perceive schizophrenic voices as intrusive, whereas Ghanaians and Indians frequently report them as friendly or playful. The American experience may partly reflect individualistic dispositions, which may make voices especially violating. This characterization echoes Sadler (2007, p. 115), a psychiatrist who explains that a patient often encounters mental illness as an “ego-alien” takeover that “envelops the person” and “penetrate[s] her inner being, her personal self.” The invasiveness Sadler describes typifies schizophrenic distress among Americans but is less common among African and Indian interviewees.

Third, these outcomes become especially interesting when read through earlier cited PP theorizing linking schizophrenia to reduced intentional binding. As a caveat, factors beyond cultural experiences of self and agency can influence schizophrenia rates and shape its course. Stress, for example, is reciprocally related to schizophrenia: patients are more prone to it, and it can exacerbate or trigger the condition (Betensky et al., 2008; Chan et al., 2015; Corcoran et al., 2002; Kemp et al., 2025; Paganin & Signorini, 2024; E. F. Walker et al., 2013). Conversely, rates often—though not always—decrease among rural populations (Padma, 2014; Pedersen et al., 2021; Vassos et al., 2012; Z. Zhan et al., 2025), and shamanistic training in African contexts where the practice is accepted may reduce toxic identification with symptoms (Luhrmann et al., 2024). An additional consideration is that group identity has been found to increase intentional binding (M. Li et al., 2019). One wonders, then, whether shamanistic training in African contexts—combined with interdependent experiences of self—might increase intentional binding while mitigating symptoms.

Here, it is also worth remembering that some Africans value autonomy yet pursue it through peer involvement that supports relational forms of selfhood (Ferrari, 2022). In such cases—and in ubuntu contexts, where privacy is often defined between groups rather than individuals (Crippen, 2021b)—an interdependent sense of self may render voices less invasive. This is at least if greater intermingling of subjectivity brings self-as-other closer to the norm, reducing problematic alienation. Such an outcome may mitigate what one of Luhrmann et al.’s interviewees described as “a hostile takeover of my mind” (Luhrmann et al., 2024, p. 451). To use Sadler’s (2007, p. 115) phrasing, a voice that “comes over” a patient may instead be experienced more as a shift in mood following a friend’s arrival (Crippen, 2025d). Something close to the latter is in fact reported in Luhrmann et al.’s (2015) interviews with non-Western people with schizophrenia.

Sadler has inspired a literature too vast to cite fully. The gist is that mental illness, while distressing in itself, becomes more so through felt loss of control and internal incursions that leave patients uncertain about who they are, thereby threatening the sense of self (Crippen, 2025d, 2025f; Dings & de Bruin, 2022; Dings & Glas, 2020; Drożdżowicz, 2023; Sadler, 2003, 2005, 2007; Tekin, 2011, 2019, 2020, 2022; Tekin & Mosko, 2015). Yet in some regions it is normal to experience oneself differently across situations within the same day (Joo et al., 2022; Nisbett, 2003; Spencer-Rodgers et al., 2009). And, as noted, both feelings of agency and the degree to which their loss is threatening vary across cultures (Bart et al., 2019; Han et al., 2022; Hornsey et al., 2019; Ji et al., 2000; Sastry & Ross, 1998; Yamaguchi et al., 2005). Additionally, those with more independent forms of selfhood are especially disturbed by internal inconsistency, which links more strongly with mental illness for them (Church et al., 2008; Cross et al., 2003; English & Chen, 2007, 2011; Suh, 2002).

Taken together, these results raise overlooked possibilities for PP understandings of schizophrenia. Against Rossetti et al.’s (2025) interpretation, measures such as intentional binding may correlate positively or negatively with schizophrenia, depending on the enculturation of one’s sense of self and personal control. 

The presented findings also challenge PP accounts that attribute schizophrenia to predictive models that poorly distinguish self-generated from externally generated actions (e.g., Ford & Mathalon, 2012; Friston, 2012; Griffin & Fletcher, 2017; Okimura et al., 2023; Rossetti et al., 2024). This is because the data suggest that people from some cultures—independently of schizophrenia diagnoses—experience a reduced distinction between self and other, between individually and situationally generated actions.

A few considerations complicate the overall picture, including both PP theorizing about schizophrenia and the proposed revisions. First, lower agency may reflect socioeconomic disadvantage (Callan et al., 2024; Daganzo & Bernardo, 2018; Esposito, 2003; Landau, 1995; K.-J. Huang, 2026), which predicts schizophrenia in industrialized nations (Corcoran et al., 2009; Werner et al., 2007). Second, schizophrenia and socioeconomic status covary with other factors such as ethnicity in the United States. 

Black Americans, for example, report less agency than whites, along with lower socioeconomic status, higher interdependence (communal orientation) but roughly equal independence (Boykin et al., 1997; Constantine et al., 2003; Gaines et al., 1997; Oyserman et al., 2002). Socioeconomic status can be a medical liability (Pedron et al., 2021). Experiences such as racism can worsen difficulties, despite sometimes fostering resilience and other limited benefits (Ben et al., 2017; Cénat, 2020; Lewis et al., 2015; Paradies et al., 2015; Priest et al., 2013; Williams & Cooper, 2019; Williams et al., 2019). Hence, although this group shows much higher schizophrenia rates than whites in the US (Anglin et al., 2021; Bazargan-Hejazi et al., 2023; Gara et al., 2019; Merritt et al., 2023; Olbert et al., 2018; Schwartz & Blankenship, 2014), disentangling the causes is challenging. Disenfranchisement may reduce socioeconomic status, which increases schizophrenia risk. At the same time, evidence suggests clinicians may too readily dismiss justified grievances about discrimination as paranoid delusions, contributing to higher diagnosis rates (Faber et al., 2023; Metzl, 2010; Moe et al., 2024; Singh & Carney-Knisely, 2024).

Corroborating this interpretation, additional evidence suggests that affective conditions such as depression are often misdiagnosed as schizophrenia in Black patients (Akinhanmi et al., 2018; Gara et al., 2019; Moe et al., 2024; Neighbors et al., 1999; Strakowski et al., 1997). Here, distress may link to persistent racism that constrains agency and socioeconomic advancement; situational depression may then be misdiagnosed as schizophrenia.

Even if schizophrenia rates remain higher after controlling for misdiagnosis, and even if Rossetti et al.’s (2025) agency measure proves valid for Black populations, this could still largely reflect other factors. For example, socioeconomic status could be key, since it affects agency and predicts schizophrenia (Callan et al., 2024; Daganzo & Bernardo, 2018; Esposito, 2003; Landau, 1995; Werner et al., 2007; Corcoran et al., 2009). This interpretation could help explain why Canada records roughly half the schizophrenia rates of the US (Han et al., 2022; Our World in Data, 2023; Z. Zhan et al., 2025), which also has higher poverty rates (Brady, 2023; Hoynes et al., 2019; U.S. Department of Agriculture, Economic Research Service, 2009; World Population Review, 2026a).

Concurrently, research suggests Canadians show lower in-group interdependence and higher independence, as well as a stronger metapersonal self (Kwantes et al., 2025). The metapersonal self extends beyond the individual and immediate social relationships to include all people, living things or even the universe (DeCicco & Stroink, 2007; Stroink & DeCicco, 2011). The founders of the concept explicitly compare it to Buddhist, Vedic and Indigenous American views (DeCicco & Stroink, 2007; Stroink & DeCicco, 2011). This connects to the earlier point that when a less bounded self is culturally normative, schizophrenia symptoms such as voice hallucinations may lose some of their threatening quality.

Revising Rossetti et al.’s (2025) PP position, one might say that cultural congruence—rather than absolute agency and self-construal per se—is the relevant factor. At the same time, PP may not be required to account for relationships between agency, self-concept and schizophrenia, as they can also be explained without it (see Crippen, 2025d).

## 11. Conclusions

This review has highlighted compelling aspects of PP. It has also criticized PP for overattributing mental illness to computationally distorting and maladaptive prediction errors. The counterexamples listed—while numerous—distill into two general sorts.

First, pathologies—though undesirable—can involve bodily attunement more than predictive gaps, as seen in the discussion of regulatory shifts among depressed elderly patients in care facilities. Here, illnesses can be distinguished from adaptive responses, e.g., slowing down under physiological stress from heart disease, COVID or depression.

Second, psychiatric conditions regularly involve anticipating bad outcomes to avert them, so that adaptive success is measured by the preservation of prediction errors. Yet a nuance remains: just as few would wish to flee a violent attack—even if the reaction is appropriately attuned—few desire to be in a situation of psychological distress.

Another conclusion in this review is that PP interpretations frequently rely on tacit normative assumptions. In fairness, such tendencies pervade psychiatry, arguably inevitably so, since a general aim of medicine is to restore normal functioning. This, in turn, is often determined through expert consensus, whether regarding acceptable cholesterol or blood pressure levels, or judgments about what counts as excessive vigilance, insufficient agency, or desirable environments and forms of selfhood.

Nevertheless, the normative dimensions of PP psychiatry remain pronounced because diagnostic thresholds are often defined in ways that strongly predispose certain patterns toward pathology. In PTSD, patients may register details (e.g., redundant clicks) that others typically miss. PP, however, typically interprets this not as heightened acuity, but as maladaptive precision-weighting: either hyperprecise threat expectations or insufficient suppression of irrelevant signals. In depression, patients may become insufficiently responsive to evidence that should otherwise subvert negative expectations. Sterzer et al. partly address concerns about explanatory flexibility by suggesting that stronger and weaker priors operate across hierarchical levels (Sterzer et al., 2018).

In fairness, stronger and weaker priors operating at the same level do not necessarily constitute a logical contradiction. In schizophrenia, PP frequently describes disrupted precision-weighting across hierarchical levels, including impaired self-monitoring, aberrant salience attribution and unstable higher-order priors. At the same time, PP also invokes overprecision, as when neutral conversations are interpreted as coded messages despite contradictory evidence. A more serious concern is that such explanatory flexibility borders on unfalsifiability, especially since mental models are not directly observable. Still, multiple philosophers of science have shown that scientific practices rely less on falsifiability than is often assumed and that this is in the interest of advancement (Feyerabend, 1993, 1999; Lakatos, 1978).

PP normative assumptions become especially visible in trauma-related conditions, where the anticipation of danger may be reasonable. Granted, one may wish to overcome debilitating fear that prevents enjoyment, say, after a mugging or upon returning to civilian life following war (Barlattani et al., 2023). But cases differ, and it may be presumptuous to tell sexual trauma survivors that they are, to requote Barrett et al. (2016, p. 9), perceiving “threats where none exist” when data suggest the following: that somewhere between 30–80% of women have experienced sexual assault or harassment with high recurrence, with rates also troubling for men (Barnes et al., 2009; National Children’s Alliance, 2025; National Sexual Violence Resource Center, 2026; Sorenson et al., 1991; H. E. Walker et al., 2019; World Health Organization, 2024). The upshot is that claims about aberrant prediction can be difficult to maintain in any simple or globally uniform way.

Western idealizations of selfhood and agency also shape PP interpretations of schizophrenia, and the implications extend beyond just that. As with Barrett et al. (2016), Pizzolla (2026) links mental illness to energy dysregulation, though with an additional claim: anticipatory mismatches may produce fatigue, weakening prediction-related processes associated with personal agency, feeding back to reinforce exhaustion, exacerbating conditions like depression. Yet an account of this kind would benefit from greater cultural sensitivity. Individualism—closely tied to Western ideas about agency—has been shown to increase sensitivity to negative social evaluation, physical pain and its neural correlates in Chinese subjects (C. Wang et al., 2014; T. Zhang et al., 2017), while heightening feelings of marginalization among diverse bicultural populations (Ferenczi et al., 2015).

Depression rates are generally higher in the West despite evidence for greater genetic vulnerability in East Asia (Chiao & Blizinsky, 2010; Giannakopoulou et al., 2021). Differences in energy regulation may be involved. One factor is diet (Chen et al., 2009). Another is BMI, which tends to correlate positively and negatively with depression, respectively, in the West and East Asia, with further rural–urban and other kinds of variation at play (Giannakopoulou et al., 2021; O’Loughlin et al., 2023). Likewise, interleukin-6—a depression marker also linked to inflammation, fat breakdown, glucose uptake and appetite—appears more strongly associated with negative emotions in Americans than in Japanese (Miyamoto et al., 2013; Ting et al., 2020).

Broadly, both ethnic and cultural groups vary considerably in their psychological and physiological experiences of mental illness (Bailey et al., 2019; Crippen, 2025a; Eisenberg et al., 2008; Hankerson et al., 2011; Kirmayer, 2001; Y. Li et al., 2023; Luhrmann et al., 2015, 2024; Marazziti et al., 2021; Petrica et al., 2025; Takahashi et al., 2021; Tanaka-Matsumi & Marsella, 1976). Ethnic and cultural variations include different responses to psychopharmaceuticals (Marazziti et al., 2021; Petrica et al., 2025; Takahashi et al., 2021). One example is ketamine (Petrica et al., 2025; Takahashi et al., 2021). PP theorists have used reactions to this drug to model psychosis in terms of aberrant precision-weighting and hierarchical prediction, also suggesting it as a therapy to loosen rigid priors and promote belief revision (Adams et al., 2013b; Bottemanne et al., 2023; Corlett et al., 2016; Haarsma et al., 2022; Harding et al., 2024; Sheldon et al., 2022). Yet rather than undermining PP, attention to cultural variability may expand the theory’s reach and clinical sensitivity across conditions.

As stated, deeply normative strains pervade psychiatry and not just PP. The *DSM-5-TR* (APA, 2022) typically requires distress for diagnosis, though such distress may partly arise from how conditions are socially framed. This may sometimes be justified, as in exhibitionistic and voyeuristic disorders, which involve distressing violations of social and legal norms. Still, these criteria remain strongly moralized.

However, in cases such as ADHD, autism and the *DSM*’s former classification of homosexuality, psychiatric judgments become equivocal or outright problematic. As argued in this review and the neurodiversity literature, distress may arise less from dysfunction than from social hostility toward certain ways of being (Armstrong, 2010; Crippen, 2025d; López-Fernández & Martínez, 2026; Timimi & Leo, 2009; Uddin, 2022). PP is especially susceptible to such hidden normativity because of its emphasis on statistical regularities, though as a newer framework it also has opportunities to avoid reproducing longstanding psychiatric excesses.

Returning to the wide explanatory reach, defenders often frame this as a PP strength. Jin et al. (2023), for example, present PP as a holistic framework integrating neuroscience, developmental psychology, emotion, trauma, social context and behavior within a unified explanatory structure. Its distinctive feature lies in translating diverse phenomena into a shared computational vocabulary. But while PP broadens what counts as relevant input, it reduces explanation to a single underlying principle: predictive inference.

At the same time, excesses appear across embodied cognition debates more generally. PP can overstate the role of internal modeling, especially where inferential constructs exceed what neuroscience can directly verify. Conversely, strongly anti-representational versions of ecological psychology and enactivism face their own difficulties. As Chemero (2013)—a prominent advocate of both—has said, it is hard to conceive of planning a dinner party without representations. Thus, while these approaches have shown that a great deal can be accounted for without recourse to representations, this differs from eliminating them altogether (Crippen, 2026b).

The unifying ambitions of PP remain theoretically attractive because they replace fragmented vocabularies with a common explanatory framework linking biological, cognitive and social processes. However, longstanding problems associated with grand unifying theories remain. Counterexamples can threaten the framework, and PP risks flattening complex lived experience into abstract informational categories. Given the complexity of mind and psychopathology, greater theoretical restraint and broader pluralistic engagement may offer favorable foundations for future research and clinical understanding.

## Data Availability

No new data generated for this article.
